# In Regard to Huang et al.

**DOI:** 10.1016/j.adro.2022.100932

**Published:** 2022-07-15

**Authors:** Kunal K. Sindhu, Brianna M. Jones, Anthony D. Nehlsen, Eric J. Lehrer, Andrew W. Smith, Jared P. Rowley

**Affiliations:** Department of Radiation Oncology, Icahn School of Medicine at Mount Sinai, New York, NY

We read with interest the recent article by Huang et al examining the impact of residency applicants’ research productivity on the Match in radiation oncology (RO). The authors showed that the number of first-author publications was associated with matching into residency programs that were ranked higher in Doximity's reputation and research output rankings. Additionally, the h-index of these applicants was associated with matching into higher ranked residency programs by research output.^1^ The h-index is a leading measure of research productivity that has been increasingly used in decisions regarding funding and hiring.^2^^,^^3^

Doximity's reputation rankings are widely used by residency applicants in the United States. However, questions have emerged regarding their accuracy and transparency.^4^, ^5^, ^6^ To further investigate the utility of these rankings in RO, we thus aimed to assess their link to the research productivity of residents at US RO residency programs.

We utilized the PubMed database to identify the first-author publications of RO residents who graduated between 2015 and 2019 that were published from the start of RO residency until three months after the completion of residency as previously described.^7^^,^^8^ We subsequently obtained the number of citations that these publications received as of October 19, 2020 from *Scopus* and grouped the publications to develop a first-author h-index for each residency program.^9^ We obtained the 2019-2020 Doximity reputation rankings for US RO residency programs from Doximity's Residency Navigator.^10^ We then assessed for a correlation between the calculated h-index ranking of RO residency programs and their corresponding Doximity reputation ranking.

Our results are shown in table 1. Among all US RO residency programs, the h-index ranking was correlated to the Doximity reputation ranking (r = 0.65). However, the correlation was considerably stronger among the top 20 programs by h-index (r = 0.73) than those ranked outside of the top 20 (r = 0.49).

**Table 1:** 

**Residency Program**	**H-index**	**2020 Doximity Reputation Rank**
Memorial Sloan Kettering Cancer Center Program	33	1
University of Texas M D Anderson Cancer Center Program	28	2
University of South Florida Morsani Program	24	55
Massachusetts General Hospital/Brigham and Women's Hospital/Harvard Medical School Program	22	3
University of Nebraska Medical Center College of Medicine Program	22	80
Yale-New Haven Medical Center Program	22	16
University of Pennsylvania Health System Program	20	5
University of Colorado Program	19	28
UPMC Medical Education Program	19	27
Washington University/B-JH/SLCH Consortium Program	17	8
Johns Hopkins University Program	16	10
Temple University Hospital/Fox Chase Cancer Center Program	16	36
Cleveland Clinic Foundation Program	15	12
Duke University Hospital Program	15	11
Emory University School of Medicine Program	15	22
Stanford Health Care-Sponsored Stanford University Program	15	4
University of Michigan Health System Program	15	6
University of North Carolina Hospitals Program	15	21
UCLA David Geffen School of Medicine/UCLA Medical Center Program	14	25
Mayo Clinic College of Medicine and Science (Rochester) Program	13	9
Sidney Kimmel Medical College at Thomas Jefferson University/TJUH Program	13	49
University of California (San Francisco) Program	13	7
University of Texas Southwestern Medical Center Program	13	23
University of Virginia Medical Center Program	13	32
University of Wisconsin Hospitals and Clinics Program	13	17
University of California (San Diego) Medical Center Program	12	13
University of Utah Health Program	11	43
MedStar Health/Georgetown University Hospital Program	10	54
New York University School of Medicine Program	10	34
SUNY Health Science Center at Brooklyn Program	10	91
Wake Forest University School of Medicine Program	10	52
Jackson Memorial Hospital/Jackson Health System Program	9	45
Ohio State University Hospital Program	9	42
University of Washington Program	9	18
Icahn School of Medicine at Mount Sinai Program	8	31
New York Presbyterian Hospital (Columbia Campus) Program	8	46
Oregon Health & Science University Program	8	29
University of Alabama Medical Center Program	8	35
University of Maryland Program	8	19
Beaumont Health (Royal Oak) Program	7	56
New York-Presbyterian Hospital (Cornell Campus) Program	7	62
University of California Davis Health Program	7	48
University of Chicago/University of Illinois College of Medicine at Chicago Program	7	15
University of Florida Program	7	14
University of Louisville School of Medicine Program	7	73
University of Texas Medical Branch Hospitals Program	7	71
Virginia Commonwealth University Health System Program	7	51
Henry Ford Hospital/Wayne State University Program	6	53
McGaw Medical Center of Northwestern University Program	6	33
Rutgers Robert Wood Johnson Medical School Program	6	38
Tufts Medical Center Program	6	72
University at Buffalo Program	6	64
University of Iowa Hospitals and Clinics Program	6	39
University of Rochester Program	6	60
Vanderbilt University Medical Center Program	6	40
Allegheny Health Network Medical Education Consortium (AGH) Program	5	74
Baylor College of Medicine Program	5	58
Case Western Reserve University/University Hospitals Cleveland Medical Center Program	5	37
City of Hope National Medical Center Program	5	57
Kaiser Permanente Southern California (Los Angeles) Program	5	66
Loma Linda University Health Education Consortium Program	5	89
Medical College of Wisconsin Affiliated Hospitals Program	5	41
Rush University Medical Center Program	5	63
Texas A&M College of Medicine-Scott and White Medical Center (Temple) Program	5	84
University of Kansas School of Medicine Program	5	70
Zucker School of Medicine at Hofstra/Northwell Program	5	24
Indiana University School of Medicine Program	4	75
Montefiore Medical Center/Albert Einstein College of Medicine Program	4	59
University of Arizona College of Medicine-Tucson Program	4	77
University of California (Irvine) Program	4	65
University of Kentucky College of Medicine Program	4	67
University of Southern California/LAC+USC Medical Center Program	4	44
Cedars-Sinai Medical Center Program	3	69
Loyola University Medical Center Program	3	30
Mayo Clinic College of Medicine and Science (Arizona) Program	3	20
Mayo Clinic College of Medicine and Science (Jacksonville) Program	3	26
National Capital Consortium Program	3	61
New York-Presbyterian Brooklyn Methodist Hospital Program	3	88
University of Mississippi Medical Center Program	3	85
Detroit Medical Center/Wayne State University Program	2	81
Medical University of South Carolina Program	2	47
SUNY Upstate Medical University Program	2	79
University of Cincinnati Medical Center/College of Medicine Program	2	50
University of Minnesota Program	2	68
University of Tennessee College of Medicine Program	2	82
University of Oklahoma Health Sciences Center Program	1	86
University of Texas Health Science Center San Antonio Joe and Teresa Lozano Long School of Medicine Program	1	76

Doximity's residency reputation ranking is calculated by asking board-certified physicians to nominate up to five programs that provide the best clinical training.^11^ This methodology thus yields rankings that are inherently subjective and, by limiting nominations to five per physician, may be less suitable for stratifying less highly ranked residency programs. While the strength of a residency program is not necessarily reflected in the research output of its residents, our data hint at this possibility. Applicants to the field of RO inclined to utilize the Doximity reputation rankings should do so with caution.
